# Why does COVID-19 disproportionately affect older people?

**DOI:** 10.18632/aging.103344

**Published:** 2020-05-29

**Authors:** Amber L. Mueller, Maeve S. McNamara, David A. Sinclair

**Affiliations:** 1 Glenn Center for Biology of Aging Research, Blavatnik Institute, Harvard Medical School, Boston, MA 20115, USA

**Keywords:** aging, cytokine storm, COVID-19, epigenetic clock, immunity

## Abstract

The severity and outcome of coronavirus disease 2019 (COVID-19) largely depends on a patient’s age. Adults over 65 years of age represent 80% of hospitalizations and have a 23-fold greater risk of death than those under 65. In the clinic, COVID-19 patients most commonly present with fever, cough and dyspnea, and from there the disease can progress to acute respiratory distress syndrome, lung consolidation, cytokine release syndrome, endotheliitis, coagulopathy, multiple organ failure and death. Comorbidities such as cardiovascular disease, diabetes and obesity increase the chances of fatal disease, but they alone do not explain why age is an independent risk factor. Here, we present the molecular differences between young, middle-aged and older people that may explain why COVID-19 is a mild illness in some but life-threatening in others. We also discuss several biological age clocks that could be used in conjunction with genetic tests to identify both the mechanisms of the disease and individuals most at risk. Finally, based on these mechanisms, we discuss treatments that could increase the survival of older people, not simply by inhibiting the virus, but by restoring patients’ ability to clear the infection and effectively regulate immune responses.

## INTRODUCTION

Severe Acute Respiratory Syndrome coronavirus 2 (SARS-CoV-2), which is responsible for the worldwide pandemic of coronavirus disease (COVID-19) originated in Wuhan, China, in late 2019 [1]. COVID-19 has so far killed more than 350,000 people, with the majority of deaths (74%) occurring in people over the age of 65 [2, 3]. Why the disease is particularly dangerous in older people is not yet known and poorly understood at the molecular level. It is clear, however, that age alone is by far the most significant risk factor for death due to COVID-19 [4, 5]. Even prior to SARS-CoV-2, human coronaviruses and influenza viruses have been known to impact older people disproportionately [6], yet therapeutic strategies to protect this fraction of the population, with the exception of vaccines, have largely failed. The severity of COVID-19 is, of course, strongly associated with comorbidities such as hypertension, diabetes, obesity, cardiovascular disease, and respiratory system diseases [2]. Whether these comorbidities contribute specifically to SARS-CoV-2 pathogenesis or whether they are primarily indicators of biological age remains an open question. For example, simple explanations for the impact of age that are based solely on co-morbidities or on a general lack of resilience in aging, for example, fail to explain why the immune system often reacts uncontrollably.

**Figure 1:**
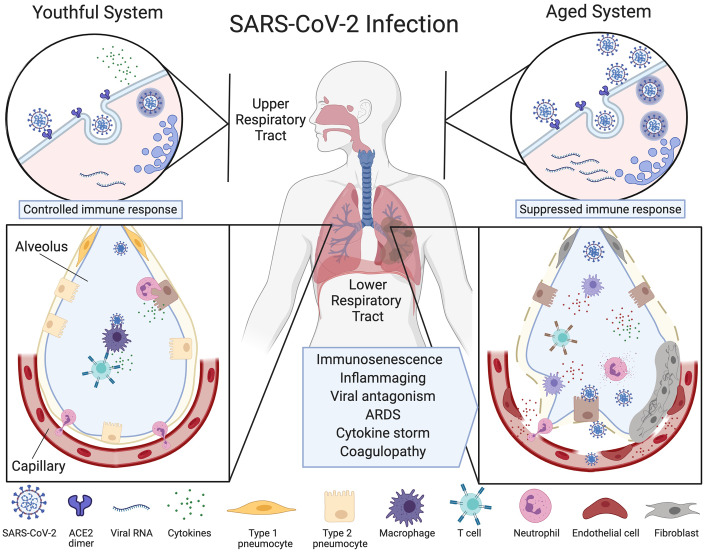
**Ineffective clearance of SARS-CoV-2 infection in the aged respiratory system.** The SARS-CoV-2 virus binds to ACE2 enzymes on airway epithelial cells in the upper respiratory tract where they are endocytosed and replicated (top left), alerting the immune system. Viruses then travel to the alveoli and infect type 2 pneumocytes which, in the youthful system (lower left), are recognized by alveolar macrophages (AMs) or dendritic cells (not pictured) that release cytokines and present antigens to T cells and other adaptive immune cells. T cells with the appropriate receptors activate other lymphocytes or directly kill infected cells, preventing the spread of the virus. Neutrophils migrate to the sites of infection to clear infected cell debris. In the aged system (top right), viral alert signals are initially slow, resulting in greater viral replication. Defective macrophages and T cells with a limited repertoire of receptors are less effective (lower right). More cells are infected, inducing high levels of inflammatory cytokine signaling. The endothelial cell lining of the capillary becomes inflamed, fibroblasts are activated, and SARS-CoV-2 viral components and cytokines enter the bloodstream. Fluid fills the alveolus, reducing lung capacity and the virus infects microvascular pericytes in other organs. A cytokine storm initiates microvasculature clotting, causing severe hypoxia, coagulopathy and organ failure. Created with BioRender.

SARS-CoV-2 is transmitted through respiratory droplets or by direct contact. Entering the nose, mouth or eyes, the virus spreads to the back of the nasal passages, where it binds to and enters via the dimerized angiotensin-converting enzyme 2 (ACE2) [7] on the surface of airway epithelial cells [8]. From there, it spreads to the mucous membranes of the throat and bronchial tubes, eventually entering the lungs where it infects type 2 alveolar epithelial cells called pneumocytes. This can lead to acute respiratory distress syndrome (ARDS), characterized by a loss of beneficial lung surfactant and an increase in oxidative stress and inflammation [9, 10] (Figure 1).

Particularly in older people, severe cases of the disease are characterized by acute lung injury and ARDS, the latter of which is typically treated by positive airway pressure with oxygen and pronation or invasive ventilation. This stage is characterized by neutrophilia, lymphocytopenia, lung consolidation, and bilateral nodular and peripheral ground glass opacities on chest X-rays. The ACE2 protein is widely expressed on the surface of both epithelial and microvascular pericytes that traverse multiple organs allowing both cell types to be infected by the virus [11, 12]. The recruitment of immune cells to sites of infection results in widespread inflammation and endothelial dysfunction in the lung, heart, kidney, and liver and brain, with prominent endotheliitis of the submucosal vessels and apoptotic bodies [11].

Even if viral loads decline in the patient, a type of cytokine release syndrome can rapidly develop, characterized by disseminated intravascular coagulation (DIC), causing liver damage, renal dysfunction, cardiovascular inflammation, coagulopathy and death [13, 14]. There are very few studies that definitely connect the known mechanisms of aging to the pathogenesis of viruses. In this perspective, we offer potential mechanistic explanations as to why COVID-19 advances in some people and not others, and especially in older patients, including differences in the immune system, glycation, the epigenome, inflammasome activity, and biological age. We also discuss therapies that may improve immunity against viral infection while enhancing the ability of older people to recover from severe COVID-19.

### The aging immune system

The ability to control viral load is one of the best prognostics of whether a patient will have mild or severe COVID-19 symptoms [15]. For the immune system to effectively suppress then eliminate SARS-CoV-2, it must perform four main tasks: (1) recognize, (2) alert, (3) destroy and (4) clear. Each of these mechanisms are known to be dysfunctional and increasingly heterogeneous in older people [16, 17]. But which tasks are most relevant to COVID-19 progression in older people is not yet clear [18].

During aging, the immune system changes in two major ways. One is a gradual decline in immune function called immunosenescence, which hampers pathogen recognition, alert signaling and clearance. This is not to be confused with cellular senescence, an aging-related phenomenon whereby old or dysfunctional cells arrest their cell cycle and can become epigenetically locked into a pro-inflammatory state in which they secrete cytokines and chemokines. The other classic immune system change during aging is a chronic increase in systemic inflammation called inflammaging, which arises from an overactive, yet ineffective alert system [19].

An abundance of recent data describing the pathology and molecular changes in COVID-19 patients points to both immunosenescence and inflammaging as major drivers of the high mortality rates in older patients. Within immunosenescence, there are defects in both the innate and adaptive immune systems. Innate immunosenescence is characterized by ineffective pathogen recognition and macrophage activation, and a reduction in natural killer (NK) cell cytotoxicity, whereas adaptive immunosenescence is characterized by thymic atrophy and accumulation of anergic memory lymphocytes. In both cases, these age-related changes are thought to be due to pathogenic, genetic, and lifestyle factors that affect the cells’ epigenetic status and the diversity of immune cells.

### The aging innate immune system

The innate immune system is the body’s first line of defense against coronaviruses. Sentinel cells, such as macrophages and dendritic cells, recognize structurally conserved viral proteins via single-pass membrane-spanning receptors called Toll-like receptors (TLRs) expressed on their cell surfaces. Defects in TLR function in innate immune cells are known to increase the severity of pneumonia in mice, especially in the context of aging and chronic inflammaging [20]. Alveolar macrophages (AMs) are mononuclear phagocytes that surveil the lungs for dust, allergens and the remnants of pathogens. When their TLRs detect an invader, AMs respond by producing type I interferons, which attract immune cells to the site of infection and present antigens to lymphocytes [21, 22]. Although AMs increase in number during aging, their plasticity to convert between pro- and anti-inflammatory states is greatly reduced [23], exemplified by a weak cytokine response after TLR activation [24] (Figure 1).

The inability of AMs in older individuals to recognize viral particles and convert to a pro-inflammatory state likely accelerates COVID-19 in its early stages, whereas in its advanced stages, AMs are likely to be responsible for the excessive lung damage. A recent study comparing immune cell composition of bronchoalveolar lavage fluid from moderate and severe COVID-19 patients showed in severe cases, macrophages were phenotypically more proinflammatory, expressing higher levels of CCR1 and CXCR2 that recruit other innate immune cells, compared to macrophages from moderate COVID-19 cases that expressed more T-cell attracting chemokines [25]. Prolonged monocyte activation is a well-known cause of severe lung injury in rhesus monkeys [26] and in cases of SARS (caused by SARS-CoV-1), higher numbers of pulmonary neutrophils and macrophages correlated with the development of ARDS and greater lung damage [27]. A decline in neutrophil activity might also be partly responsible because, during aging, these cells progressively lose their ability to migrate to sites of infection and kill infected cells [28, 29]. NK cells, a major component in innate immunity with potent cytotoxic activity, are an unlikely cause of COVID-19 severity. Their numbers are relatively stable during aging [30] and in a mouse model of SARS, they were not necessary for normal viral clearance [31]. To discern which of these cell types play the most destructive roles, more detailed analyses of COVID-19 patient autopsy tissue will be needed.

Additionally, the production and diversity of mucins, protective glycoproteins found in mucosal barriers throughout the body, also change in aging [32, 33], although their role in immunity against coronaviruses in humans is understudied.

### The aging adaptive immune system

**Figure 2:**
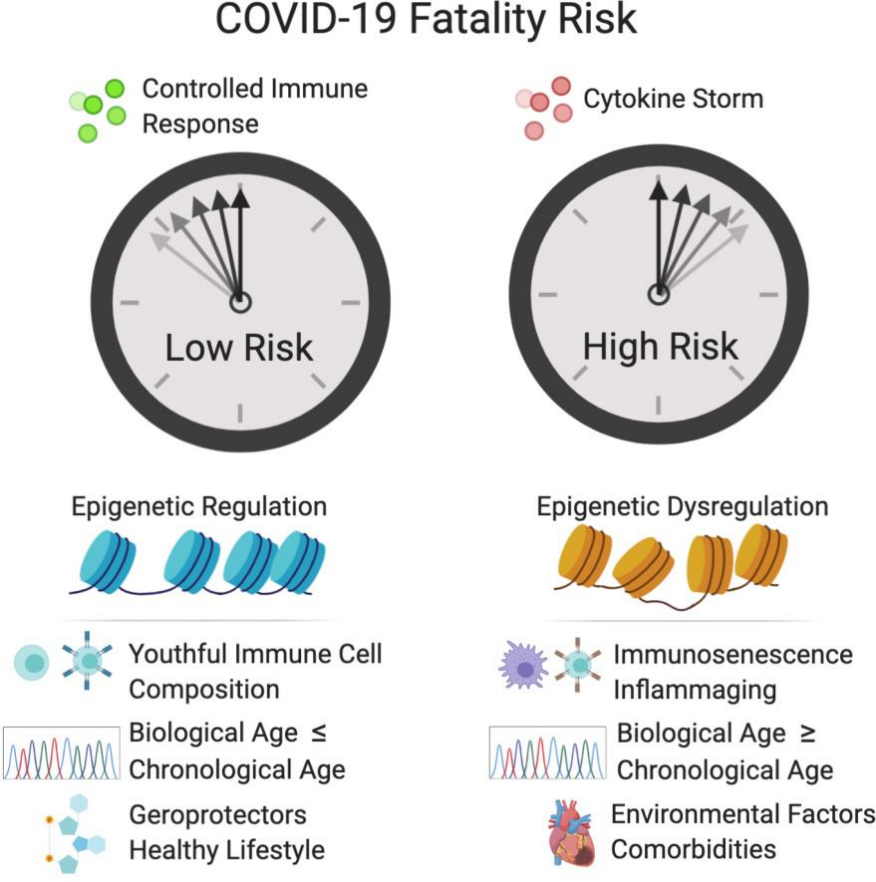
**Factors that increase the fatality risk of COVID-19.** Epigenetic dysregulation, immune defects, advanced biological age, and other factors increase the risk of cytokine storm and COVID-19 fatality. Tightly controlled activation of the innate immune system is essential for viral recognition and clearance. Cytokine storm is the result of sustained activation of the inflammatory signaling cascade and can result in hypercoagulation in small blood vessels, which leads to tissue damage, DIC and multi-organ failure. Inflammaging and immunosenescence contribute to the development of cytokine storm. D-dimer, a fibrin degradation product and prognostic of disseminated intravascular coagulation (DIC), and elevated levels of the cytokine, IL-6, are associated in the clinic with increased fatality. Epigenetic dysregulation of the immune system and of the renin-angiotensin system (R)AS may increase fatality risk. A variety of biological clocks have been shown to predict human health and longevity more accurately that chronological age. An individual with a biological age greater than their chronological age is thought to be undergoing accelerated aging, which may increase the risk of COVID-19 fatality. Individuals with comorbidities such as cardiovascular disease, diabetes, obesity and COPD, are at greater risk for COVID-19 fatality. Conversely, individuals who live healthy lifestyles and consume geroprotectors such as metformin, resveratrol and NAD^+^ boosters may have a decreased risk of fatality. Created with BioRender.

Immunosenescence of the adaptive immune system is also a likely factor that determines whether a patient progresses to severe COVID-19 (Figure 2). Situated just above the heart, the thymus – a primary lymphoid organ and the site of T cell development and maturation of early thymic progenitors from the bone marrow – is one of the first tissues to experience aging. By age 65, the thymus is on average ~40% its original size [34], coincident with activation of the inflammasome component NLRP3 and Caspase-1, a pro-apoptotic protease [35, 36]. A build-up of intrathymic adipocytes further reduces thymic cellularity and deteriorates the thymic microenvironment. Thymic atrophy also contributes to a reduction of naïve T cells and an accumulation of memory lymphocytes, resulting in defective immunosurveillance and an exhaustion of B cells, cytotoxic T cells, and helper T cells [37]. Other common effects of aging on the adaptive immune system include a decline in the production of fresh naïve T cells, a less expansive T cell receptor (TCR) repertoire, T cell metabolic dysfunction, and weaker activation of T cells [38, 39]. Clonal populations of CD8^+^ T cells expand during aging, limiting their diversity, whereas CD4^+^ T cells retain fairly diverse TCRs [40] and, instead, suffer activation deficits [39].

Interestingly, one study found that supercentenarians – defined as adults over 110 years old – tend to have an unusual population of cytotoxic CD4^+^ T cells whose activation doesn’t decline with age and can take on the effector functions usually performed by CD8^+^ T cells [41]. This T cell behavior may explain why some older people, even some people over 100, are able to survive COVID-19. Measuring the repertoire and frequency of TCRs in patients from a spectrum of ages and disease severity should be performed to determine if a loss of T cell diversity is a reason why SARS-CoV-2 viral loads tend to spike in older people but not the young.

Not only does the repertoire of T cells decline in aging, so do their numbers. Those over 60 years old increasingly have low T cell numbers, a condition known as lymphopenia [42]. Because T cells express very low levels of ACE2, the lymphopenia in COVID-19 patients is unlikely to be caused by direct SARS-CoV-2 infection [43], as in the case for HIV. One proposed cause of the T cell paucity is an exhaustion of the immune system driven by repeated exposures to viruses over one’s lifetime [42, 44, 45]. This hypothesis is based on several studies that tracked the morbidity and mortality of people over 60 who had been chronically infected with human cytomegalovirus (CMV) [46, 47]. Cycles of CMV reemergence were associated with vast immune system remodeling, including a pronounced exhaustion of CD8^+^ T cells that was more predictive of all-cause mortality than chronological age. Other studies indicate that T cell depletion is due to the cumulative exposure to many different pathogens and lifestyle factors, not CMV alone [46, 48]. At the chromosomal level, a major cause of immune exhaustion is telomere shortening in viral-specific memory CD8^+^ T cells, which induces cellular senescence, a state of cell cycle arrest and hyper-inflammation that prevents expansion upon re-infection [49]. The fact that in the most severe COVID-19 cases bronchoalveolar CD8^+^ T cells appear to have reduced expansion capability [25] and peripheral blood T cells express high levels of the immune-exhaustion marker PD-1 [42] make this theory plausible.

B cells – adaptive immune cells which produce antibodies in response to coronavirus antigens [21] – are also less diverse and less responsive in aging [50, 51]. While total B cells numbers do not decrease in aging, memory B cells accumulate and naïve B cells are depleted, which may lead to loss of diversity of the B cell repertoire, although this has not yet been definitively demonstrated in humans [51]. Changes in IgG glycosylation patterns, however, have been shown to strongly associate with age and inflammation, and predict age-associated disease development [52]. In particular, IgG N-glycans appear to be the most predictive of biological aging, however B-cell intrinsic and extrinsic regulation of glycosylation in aging require further study.

Due to a lack of sun exposure and decreased production of vitamin D, about half of all older people have a deficiency in this vitamin [53], which reduces the efficacy of both adaptive and innate immune responses and increases the risk of infection [54]. Vitamin D levels in older people are correlated with preserved features of immunity such as the CD4^+^/CD8^+^ ratio and lower levels of pro-inflammatory cytokines after stimulus [54, 55]. Although not all studies see a benefit of vitamin D supplementation on the risk or duration of lower respiratory infections [56], the majority have, especially in those with an antibody deficiency or increased susceptibility to respiratory tract infections [57, 58]. A recent meta-analysis of 25 randomized, placebo-controlled trials concluded that vitamin D supplementation prevented about 20% of acute respiratory infections [59]. As such, some health professionals have recommended vitamin D supplementation for older people in general and especially for aged-care residents and critically ill patients as a strategy for improving chances of COVID-19 survival.

### Increased inflammation and cytokine storms in the aged

During the course of COVID-19, older patients can reduce their viral titers, only to rapidly descend into a state of shock involving hyperactivation of the immune system and hypercoagulation in small blood vessels [42, 60]. This rapid and uncontrolled inflammatory signaling cascade typically occurs in the later stages of infection. Known as a “cytokine storm,” it exacerbates the dyspnea and hypoxemia, and triggers inflammation in major tissues such as the lungs, kidneys, heart, liver and brain. Cytokine storm syndrome is defined as life-threatening organ dysfunction caused by a maladaptive host response to an infectious trigger [61]. The resulting vascular inflammation is emerging as the cause of complement-associated microvascular injury and thrombosis in severe COVID-19 cases [62]. The initial trigger for cytokine storm is not yet known but it likely involves the immune system’s detection of a large quantity of viral antigens released by dying cells. Why older people are particularly prone to cytokine storms is also unclear.

The cytokine profiles of late-stage COVID-19 patients are similar to patients with secondary haemophagocytic lymphohistocytosis, a type of cytokine storm that can be triggered by systemic viral infection, including increased levels of interleukin (IL)-2, IL-6, IL-7, C-reactive protein (CRP), granulocyte-colony stimulating factor (GCSF), interferon-γ inducible protein 10 (IP-10), monocyte chemoattractant protein-1 (MCP-1), macrophage inflammatory protein 1-α (MIP1-α) and tumor necrosis factor-α (TNF-α) [45, 63, 64].

Even more predictive of death than serum cytokine profiles is an increase in the fibrin degradation product D-dimer, released from blood clots in the microvasculature, and a prognostic for DIC [9]. As such, D-dimer is now widely regarded as a key indicator of the severity of late-stage COVID-19. D-dimer levels naturally increase with age, most likely reflecting a higher basal level of vascular inflammation [65], which could predispose patients to severe COVID-19. It would, therefore, be informative to know if pre-cytokine storm levels of D-dimer levels could predict who is likely to develop a cytokine storm.

In cytokine storms, high levels of IL-6 cause vascular endothelial cells to secrete fibrin, which causes DIC. In the lung, this may underlie the hypoxemia seen in patients with seemingly functional lungs. If left untreated, clots leach additional clotting factors from the bloodstream, increasing the risk of bleeding (coagulopathy) and multi-organ failure. Drugs such as tocilizumab (Actemra), which block IL-6 receptor activity, are currently being used in patients in advanced stages [66].

One in two fatal cases of COVID-19 experience a cytokine storm, 82% of whom are over the age of 60 [67]. Though there may be many simultaneous triggers of the storm, abundant evidence indicates that inflammaging is a major driver, exacerbated by obesity, poor diets and oral health, microbial dysbiosis, and sedentary lifestyles [68, 69]. For example, in rodents, inflammaging increases the risk of cytokine storm syndrome [70] and, in humans, age correlates with higher basal circulating levels of pro-inflammatory cytokines including IL-6, TNF-α, IL-1α and CRP [71, 72].

A central player that could help explain the predisposition to cytokine storms is NLRP3, the major protein component of the inflammasome. During aging, there is a steady increase in the abundance and activity of NLRP3 in immune cells, including AMs of the lung which, upon chronic stimulation, contribute to pulmonary fibrosis [73]. NLRP3 inflammasome activation requires two steps, the first of which is the priming step, induced by TLRs or tumor necrosis factor receptor activation. This leads to the activation of NF-κB and promotes the expression of NLRP3, pro-IL-1β, and pro-IL-18. The second step, also called the activation step, is triggered by a range of stimuli that emerge during infections, such as tissue damage, nucleic acids, and invading pathogen proteins [74].

In older individuals, NLRP3 may be poised for hyperactivation by SARS-CoV-2 antigens. NLRP3 activity is under the direct control of sirtuin 2 (SIRT2), a member of the NAD^+^-dependent sirtuin family of deacetylases (SIRT1-7) [75]. During aging, NAD^+^ levels decline, reducing the activity of the sirtuins [76]. Old mice, especially those deficient in SIRT2, have decreased glucose tolerance and increased insulin resistance [77]. This decline, exacerbated by COVID-19, might promote hyperactivation of NLRP3 and the trigger cytokine storms in COVID-19 patients [14]. Maintaining NAD^+^ levels may therefore alleviate COVID-19 symptoms, a possibility supported by recent data showing that SARS-CoV-2 proteins hyperactivate poly-ADP-ribose polymerases PARP9, -10, -12, and -14 and deplete cellular NAD^+^ [78]. Additionally, NAD^+^ precursors lower inflammation in human subjects [79, 80].

Mechanisms of infection in other coronaviruses support the hypothesis that NLRP3 activation is a trigger of cytokine storms in the aged. The SARS-CoV-1 ORF3a protein, for example, is a potent activator of pro-IL-1β gene transcription and protein maturation, the two main signals required for activation of NLRP3 [81]. In macrophages, SARS-CoV-1 ORF8b robustly activates the NLRP3 inflammasome by interacting directly with the Leucine Rich Repeat domain of NLRP3 in cytosolic dot-like structures [82], suggesting another two-step model, in which inflammaging and the NLRP3 basal overactivation is the first step and SARS-CoV-2 antigen-mediated hyperactivation is the second step that triggers a cytokine storm.

In chronic diseases, hyperactivity of the inflammasome plays a dominant role in the development of type 2 diabetes and other age-related diseases [83]. Indeed, in older adults, the upregulation of two inflammasome-related gene sets correlate with increased risk of hypertension, metabolic dysfunction, oxidative stress and mortality [84]. Individuals over the age of 85 that expressed lower levels of these inflammasome modules were less likely to die within seven years [84]. Taking together, the known effects of coronavirus proteins on NAD^+^, NLRP3, and the two stages of inflammasome activation, these data provide a plausible explanation as to why co-morbidities positively correlate with cytokine storms and fatality in COVID-19 patients.

After age and hematological cancers, obesity is the next major risk factor for COVID-19 fatality, similar to type 2 diabetes [85]. Obesity is well known to increase the activity of NLRP3 and stimulate low grade inflammation in mice, including higher levels of serum chemokines, and lower neutralizing antibodies and effector memory T cells during a viral infection [86]. Accordingly, this may help explain why obesity is associated with lower survival in COVID-19, SARS-CoV-1 and MERS-CoV infections, and why obesity-related human diseases such as cardiovascular disease, chronic kidney disease, and diabetes, predispose patients to cytokine storms (Table 1) [87–89]. In addition, by causing the endothelium of the microvasculature to become leaky, obesity and type 2 diabetes, may increase the ability of SARS-CoV-2 to infect surrounding pericytes that appear to express ACE2 at levels far greater than surrounding cells [12].

**Table 1:** Risk factors for adverse outcomes in human coronavirus infections.

**Risk factor**	**Virus**	**References**
Advanced age	SARS-CoV-2, SARS-CoV-1, MERS	[4, 175–181]
Cardiovascular disease, hypertension and coronary artery disease	SARS-CoV-2, SARS-CoV-1, MERS	[4, 176, 179, 181–184]
Diabetes	SARS-CoV-2, SARS-CoV-1, MERS	[4, 176, 182, 183, 185–188]
Obesity	SARS-CoV-2, SARS-CoV-1, MERS	[4, 182, 183, 189]
Male Sex	SARS-CoV-2, MERS	[4, 176, 178]
Respiratory diseases	SARS-CoV-2, MERS	[4, 176, 181]
Kidney disease	SARS-CoV-2, MERS	[4, 176, 187, 190]
Immunological disorders	SARS-CoV-2	[4, 175]
Cancer	SARS-CoV-2, SARS-CoV-1	[4, 179]
Other factors	SARS-CoV-2, SARS-CoV-1	[4, 179, 180, 186, 191]

### Epigenetic changes with age

The dysregulation of the epigenome and resulting changes in gene expression during aging are strongly implicated as biomarkers, and potentially underlying causes, of chronic disease states and of aging itself. The “relocalization of chromatin modifiers” theory of aging postulates that symptoms of aging and the loss of resilience are a result of a lifetime accumulation of epigenetic changes [90, 91]. These changes may be caused, in part, by the redistribution of chromatin factors, such as the nuclear proteins SIRT1/6/7, HDAC1 and PARP1 away from regular loci to sites of dsDNA break repair, then back again, causing epigenetic “noise” to accumulate, which may iteratively erase cellular identity [90–94]. This process is thought to manifest as DNA methylation changes that set the pace of the biological clock in tissues and in hematopoietic cells [95, 96].

There is an abundance of evidence indicating that age-related changes to the host’s epigenome compromise immune cell composition and function [97] and negatively impact viral defenses [98, 99], including adaptive immune memory [100, 101]. Coronaviruses are known to mediate epigenetic alterations, potentially accelerating the rate that the immune system ages. MERS-CoV, for example, antagonizes host antigen presentation by altering DNA methylation, a mark that silences genes encoding major histocompatibility complexes [102]. Similarly, SARS-CoV-1 changes histone methylation and long non-coding RNAs, which is accompanied by the activation of interferon-response genes [103]. Measuring the DNA methylation age of immune cells and other blood cell types before, during, and after infection could help elucidate both how the aged epigenome impacts disease severity and how the virus alters the aged epigenome.

The vulnerability of the aged to SARS-CoV-2 may also have to do with the effects of the epigenome on viral entry, which is initiated by physical interaction between the viral spike glycoprotein receptor and the ACE2 cell surface protein [104]. While genetic differences in ACE2 are being pursued as a cause of COVID-19 severity [105], there is little attention being paid to epigenetic differences. In humans, ACE2 is ubiquitously expressed in epithelial tissues of the body, most highly in alveolar epithelial cells and enterocytes of the small intestine [106]. ACE2 is regulated in the body transcriptionally, post-transcriptionally, and post-translationally [107], although its role and regulation in COVID-19 is still poorly understood.

In both mice and rats, ACE2 expression decreases with age and is associated with an increase in aortic fibrosis and inflammation [108, 109]. In healthy human lungs, ACE2 expression does not appear to change with age [110]. Even though ACE2 is more highly expressed in the lungs of cigarette smokers [111]. A meta-analysis of COVID-19 deaths, however, did not identify smoking as a significant risk factor [4]. ACE2 promoter hypomethylation in lymphocytes correlates with transcriptional activation in patients with lupus [112], implying that transcription of ACE2 is controlled by methylation, although this mechanism has not been systematically investigated. It is known, however, that methylation at one of seven CpGs in the ACE2 promoter decreases with age and these CpGs are bordered by long-range promoter-enhancer contacts that may change over time [113]. Bisulfite sequencing of the ACE2 gene paired with transcriptomic and four-dimensional chromatin analyses will be necessary to understand if there is a causal relationship between promoter methylation, ACE2 expression, and disease outcome.

The elucidation of SARS pathogenesis is complicated by the fact that ACE2 is also part of the renin-angiotensin system (RAS) that regulates immunity, fibrosis, blood pressure, and metabolism. ACE2 counteracts vasoconstriction caused by angiotensin converting enzyme (ACE) by cleaving its product, angiotensin II. Most likely due to its role in vasodilation and reducing inflammation, ACE2 partially protects against sepsis-induced- and SARS-induced severe acute lung injury in mice [114, 115] and asthma-induced airway inflammation in rats [116]. Changes in DNA methylation during aging are known to affect the RAS [14, 117, 118]. Analysis of ACE2 gene expression in the lungs of COVID-19 patients with pulmonary arterial hypertension and chronic obstructive pulmonary disease found a correlation between ACE2 expression and COVID-19 severity [111]. Thus, age-related dysregulation of ACE2 could explain why age is such a risk factor for COVID-19 complications and why cardiovascular disease and hypertension predispose patients to develop a more aggressive form of COVID-19.

The effects of ACE inhibitors, used commonly beyond middle age to control blood pressure, are generally believed to be neutral in COVID-19 [119, 120]. Due to their opposing roles in the RAS, ACE2 expression appears to increase when ACE is inhibited, likely providing a yet unknown protective function [121]. Inhibiting ACE2 expression or blocking ACE2 accessibility could prevent viral entry but may lead to vasoconstriction and hypertension. Instead, the most promising ACE2-targeted therapeutic strategy is to infuse human recombinant soluble ACE2 into the airway or bloodstream to bind the SARS-CoV-2 spike glycoprotein receptor, preventing it from binding ACE2 on host cell surfaces [122] and slowing cell infection rates.

### Sirtuins and NAD^+^

The sirtuins are a family of NAD^+^-dependent lysine deacylases that control numerous aspects of stress resistance and pathogen defenses. SIRT1 is a nuclear histone deacetylase that suppresses viral replication and chronic inflammation [123]. By binding to the promoter region of ACE2, SIRT1 upregulates transcription under conditions of cell stress [124]. During aging, and perhaps particularly during the course of COVID-19, levels of NAD^+^ decline. This is likely due to increased NAD^+^ consumption by the CD38^+^ glycohydrolase [125] and increased transcription of the poly-ADP-ribosyl transferases, PARP9, PARP10, PARP 12 and PARP14 in mice and humans infected with SARS-CoV-2 [78]. Coronaviruses also possess an ADP-ribosylhydrolase that further depletes NAD^+^, apparently to disrupt cell signaling, DNA repair, gene regulation and apoptosis [14, 126, 127].

By negatively regulating activity of NLRP3, SIRT1 and the related protein SIRT2, seem to play key roles in suppressing acute lung inflammation during sepsis [75]. Mice lacking SIRT1, for example, display aggravated inflammasome activation, with increased production of lung proinflammatory mediators, including intercellular adhesion molecule 1 (ICAM-1) and high-mobility group box 1 (HMGB1), and a dramatic reduction of lung claudin-1 and vascular endothelial-cadherin expression [128]. Further, as a result of NAD^+^ depletion in mouse models of uncontrolled diabetes, DNA repair is blunted leading to pulmonary inflammation, senescence and fibrosis [129], which could explain why diabetics are more susceptible to COVID-19. SIRT1 also attenuates the acute inflammatory response through deacetylation of H4K16 in the TNF-α promoter [130]. Another nuclear sirtuin, SIRT6 attenuates NF-kB signaling by deacetylating H3K9 [131]. Thus, a decline in NAD^+^ and the known mis-localization of SIRT1 and SIRT6 across the genome during aging [90, 132], could be major contributors to the age-dependency of COVID-19 symptoms. As such, NAD^+^ precursors, such as NMN and NR [133], have been suggested as possible treatments for COVID-19, especially in older people [78]. Clinical studies are needed to determine if NAD^+^ supplementation would benefit in the early stages of SARS-CoV-2 to reduce replication or if NAD^+^ treatment during acute COVID-19 can hasten recovery.

### Biological clocks

Over the past decade, a variety of biological clocks have been developed to predict human health and longevity more accurately than chronological age, including those based on DNA methylation patterns [134–136], inflammaging [137], gene expression patterns [138], frailty [139, 140], serum proteins [141], and IgG glycosylation [142–144]. Given that these clocks provide a quantitative measure of the rate of aging of an individual and their overall resilience, biological clocks may be useful for identifying at-risk populations and for predicting, within those populations, who will most likely progress to severe COVID-19.

### Epigenetic clocks

Estimates based on twin studies place the contribution of non-genetic factors on predicted COVID-19 phenotype at 50% [145] and on total disease burden in old age at approximately 80% [146]. Indeed, lifestyle factors that affect the epigenome such as calorie intake may increase the susceptibility to COVID-19. Epigenetic age is greater than chronological age in various disease contexts and lower in long-lived humans, providing strong evidence that epigenetic age reflects biological age [134, 147]. Age-associated changes to the epigenome have profound effects on the immune system, including T cell function, cytokine production and macrophage pattern recognition. DNA methylation is believed to set the pace of the aging clock in several mammalian tissues, including hematopoietic cells of the immune system [95, 96]. Epigenetic clocks that measure DNA methylation at specific CpG sites are the most widely used measure of biological age and disease susceptibility [134, 147]. Restoration of the thymus using a drug cocktail of metformin, growth hormone and dehydroepiandrosterone led to the reversal of features of immunosenescence, specifically increasing naïve T cells and a decreasing senescent PD-1^+^ T cells, along with the reversal of the epigenetic clock by about 1.5 years [96]. Epigenetic age may be a better biomarker than chronological age in predicting how variation in lifestyle factors and age-associated comorbidities increase susceptibility to COVID-19 and may also help determine if COVID-19 infection accelerates epigenetic age. We hope to test both by measuring the DNA methylation ages of peripheral blood samples from thousands of COVID-19 patients and correlating methylation age measurements with clinical outcomes.

### Glycosylation clocks

Changes in glycosylation during aging may also predispose older individuals to severe COVID-19 [148]. Glycosylation is the enzymatic process by which carbohydrates called glycans, such as sialic acid, mannose and fucose, are covalently attached to proteins or lipids, typically on the cell surface or in the bloodstream. An individual's repertoire of glycans – a notable example being the type of N-glycans attached to immunoglobulins [149] – changes with age and environmental factors, such as smoking and poor diet [148]. The type of glycans attached to IgGs affects their pro- and anti-inflammatory properties [150]. Decreased galactosylation of IgGs is associated with central adiposity [151] and inflammaging in the context of diabetes [152]. Biological clocks based on IgG glycosylation are able to predict chronological age within 10 years, and can be improved by inclusion of clinical parameters [144]. Thus, changes to the glycome with age could serve both as an indicator of biological age and could potentially predict COVID-19 severity.

Aging also changes the glycome via non-enzymatic glycation, by which reducing sugars circulating in extracellular compartments covalently bind to proteins and lipids to form advanced glycation end products (AGEs). AGEs are present in large quantities in the Western diet, and greater consumption of dietary AGEs increases serum TNF-α [153]. AGEs tend to accumulate under hyperglycemic conditions and contribute to the pathology of many age-related disease such as type 2 diabetes and obesity [154]. AGEs may increase COVID-19 severity in the aged by inhibiting the NLRP3 inflammasome during the early stages of viral infection [155] when the inflammatory program is activated by the SARS-CoV-1 3a protein [156]. AGEs also play a role in activating pro-coagulation pathways [154], potentially contributing to the DIC observed in COVID-19 patients.

Glycosylation patterns specific to older people may also impact viral entry. The SARS-CoV-2 spike protein is heavily glycosylated [157], modifications that are highly conserved between coronaviruses. SARS-CoV-2 shares 20 out of 22 of glycosylated N-linkages with SARS-CoV-1 [157]. In the case of the human influenza virus, variation in sialic acid structures on the surface of cells lining the upper and lower respiratory tracts dictates tropism and age-dependent binding efficiency of the virus [158] but how changes in the coronavirus spike protein during aging might affect viral transmission and pathogenesis is not yet known. If we are to use glycation as a prognostic marker for COVID-19, it will be necessary to map the glycome in hundreds of patient samples with varying degrees of COVID-19 severity, including asymptomatic individuals.

### Immune clocks

Between individuals, heterogeneity of the immune system increases during aging [18] and may explain differences in susceptibility to infectious diseases. A biological clock based on the immune system called IMM-AGE was recently developed that predicts all-cause mortality in older adults more accurately than even DNA methylation clocks [137]. IMM-AGE overcomes the limitation of inter-human immune heterogeneity by tracking immune cell frequencies and gene expression changes longitudinally within individuals and then computationally predicting how an individual’s homeostatic immune state changes over time. Though individuals exhibit variation in immune cell-type composition, these changes fall into three stages that converge on a common "attractor point" that correlates with age and is indicative of overall physiological resilience [137]. In this way, IMM-AGE measures the entropic relationship between age and immune system remodeling, the rate of which can predict survival. Because IMM-AGE is even able to capture and predict the effect of inflammaging on the cardiovascular system, and because COVID-19 fatality is so closely tied to cardiovascular disease and inflammaging, this clock may prove to be the most accurate at identifying COVID-19-susceptible individuals. More studies are still needed to determine if and how viral infections alter these and other biological clocks, and whether variation in biological age predicts COVID-19 severity.

### Geroprotectors to improve immunity

Advanced age is by far the greatest risk factor for COVID-19 fatality, independent of underlying co-morbidities [4]. This striking fact has led many researchers to speculate whether molecules that target aging itself, called geroprotectors, could be used to combat infections in older people [5, 159]. Primarily via its regulation of cellular metabolism, the mammalian target of rapamycin (mTOR) signaling pathway controls several immune functions such as antigen presentation, immune activation, differentiation, and cytokine production [160, 161]. Low dose mTOR inhibitors exhibit a hormetic effect in older people, seemingly improving immunity and reducing rates of infection [162, 163]. People over 65 years old who took mTOR inhibitors for six weeks responded more robustly when challenged with an influenza vaccine and showed reduced levels of the T-cell exhaustion marker PD-1 [162]. In a similar clinical trial, protection from infection and an increase in anti-viral gene expression was observed even a year after the 6-week course of mTOR complex 1 (mTORC1) inhibitors [163], though the result was not reproduced in a Phase 3 trial.

Metformin, a blood glucose lowering geroprotector that activates 5’AMP-activated protein kinase (AMPK) and inhibits the mTOR pathway, has also been suggested as a possible drug to combat severe SARS-CoV-2 infection in older people. In addition to its potential insulin-sensitizing antiviral effect [164], metformin confers a myriad of anti-aging benefits including improving mitochondrial metabolism, decreasing inflammatory cytokines, protecting against genomic instability and decreasing cellular senescence [165], which may bolster the aging body’s resistance to COVID-19. Results from the ongoing Targeting Aging with MEtformin (TAME) clinical trials and others should reveal whether these anti-aging drugs are protective against SARS-CoV-2 infection [165, 166].

### Where do we go from here?

**Figure 3:**
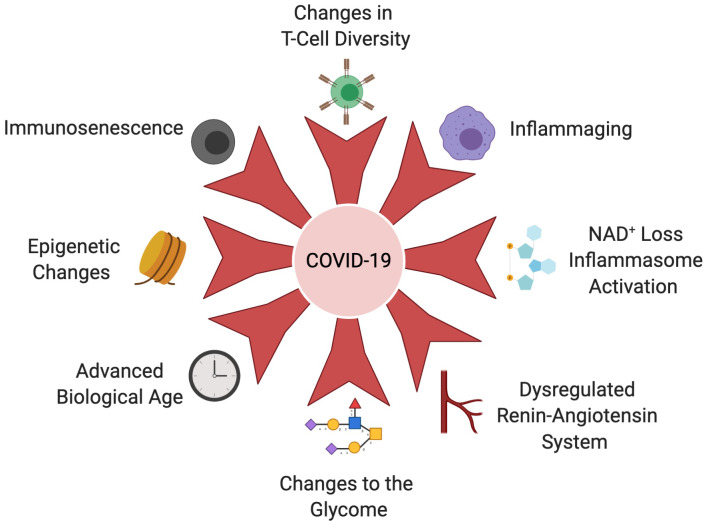
**Age-related changes that increase COVID-19 susceptibility.** The aging immune system undergoes immunosenescence, T-cell diversity alterations and chronic activation of the innate immune system, known as inflammaging. These hallmarks of the aging immune system cripple the body’s ability to clear the SARS-CoV-2 virus, initiate and sustain cytokine storms, than cause acute organ injury, DIC and multi-organ failure. An age-associated decline in NAD^+^ results in derepression of NLRP3 and inflammasome in older people, further exacerbating the cytokine storm. Coronaviruses also possess an ADP-ribosylhydrolase that further depletes already-low NAD^+^ levels in older people. Leveling of the epigenetic landscape during aging results in changes in immune cell composition and function that decrease the immune system’s ability to mount a response to infection. Epigenetic dysregulation of ACE2 may also impact increased viral loads in older people. Dysregulation of the RAS during aging and in the context of age-associated disease, such as cardiovascular disease, hypertension, COPD and obesity, contributes to severity of COVID-19 infection. The glycome which controls a variety of immune signaling pathways changes during aging and in the context of metabolic diseases. For example, decreases in IgG galactosylation contribute to chronic inflammation. Biological clocks that measure different biomarkers of biological age may explain increased COVID-19 susceptibility more accurately than advanced chronological age. Created with BioRender.

Why SARS-CoV-2 infections are more severe and fatal in the aged is not known, but viable hypotheses are emerging that include changes to the immune cell repertoire, the epigenome, NAD^+^ levels, inflammasome activity, biological clocks, and covalent modifications of human and viral proteins (Figure 3). Much remains to be elucidated still. Besides understanding the basis of the cytokine storms and coagulopathy, it is not known why SARS-CoV-2 so easily damages such a broad array of tissues in older people but rarely in the young. Nor is it clear whether older people develop stronger or weaker functional immunity during seroconversion, or how long their protection will last compared to younger people. In the aged, immune responses to vaccination are also often weak or defective [18, 167, 168], whereas autoimmunity increases [169]. Therefore, in designing vaccines against SARS-CoV-2, it will be important to consider that older people may not respond as well to vaccines as young people. Studies that follow the long-term consequences of SARS-CoV-2 infection in older people will also be critical to understand the long-term health consequences of COVID-19 pathology, such as fibrosis and scaring of the lungs, micro-ischemic events, cardiopulmonary dysfunction, and neuropsychological disability [170]. These could significantly reduce viral resistance and lifespan in older and middle-aged people who recover from severe cases of COVID-19. The most exciting and potentially impactful technologies to combat COVID-19 and other viral pandemics are those that activate the body’s defenses against aging [5, 166]. Eventually, with advances in the field, it may even be possible to reverse the age of cells and tissues [171–174] so that high-risk older individuals can respond to viral infections as though they were young.
